# Gene model for the ortholog of *S6k* in *Drosophila yakuba*

**DOI:** 10.17912/micropub.biology.001018

**Published:** 2024-12-18

**Authors:** Grace Keirn, Cole A. Kiser, Leon F. Laskowski, Jordan Hensley, Rachel Mortan, Thuy Nguyen, Shallee T. Page, Charles Du, Jeroen T. F. Gillard, Anya Goodman, James J. Youngblom, Chinmay P. Rele, Laura K Reed

**Affiliations:** 1 The University of Alabama, Tuscaloosa, AL USA; 2 University of St. Francis, Joliet, IL USA; 3 California State University Stanislaus, Turlock, CA USA; 4 California Polytechnic State University, San Luis Obispo, CA USA; 5 Franklin Pierce University, Rindge, MA, USA; 6 Montclair State University, Montclair, NJ USA; 7 California State University, Bakersfield CA USA

## Abstract

Gene model for the ortholog of *Ribosomal protein S6 kinase* ( *S6k* ) in the Dyak_CAF1 Genome Assembly (GenBank Accession: GCA_000005975.1) of *Drosophila yakuba* . This ortholog was characterized as part of a developing dataset to study the evolution of the Insulin/insulin-like growth factor signaling pathway (IIS) across the genus *Drosophila* using the Genomics Education Partnership gene annotation protocol for Course-based Undergraduate Research Experiences.

**Figure 1. Genomic neighborhood and gene model for S6k in Drosophila yakuba .**
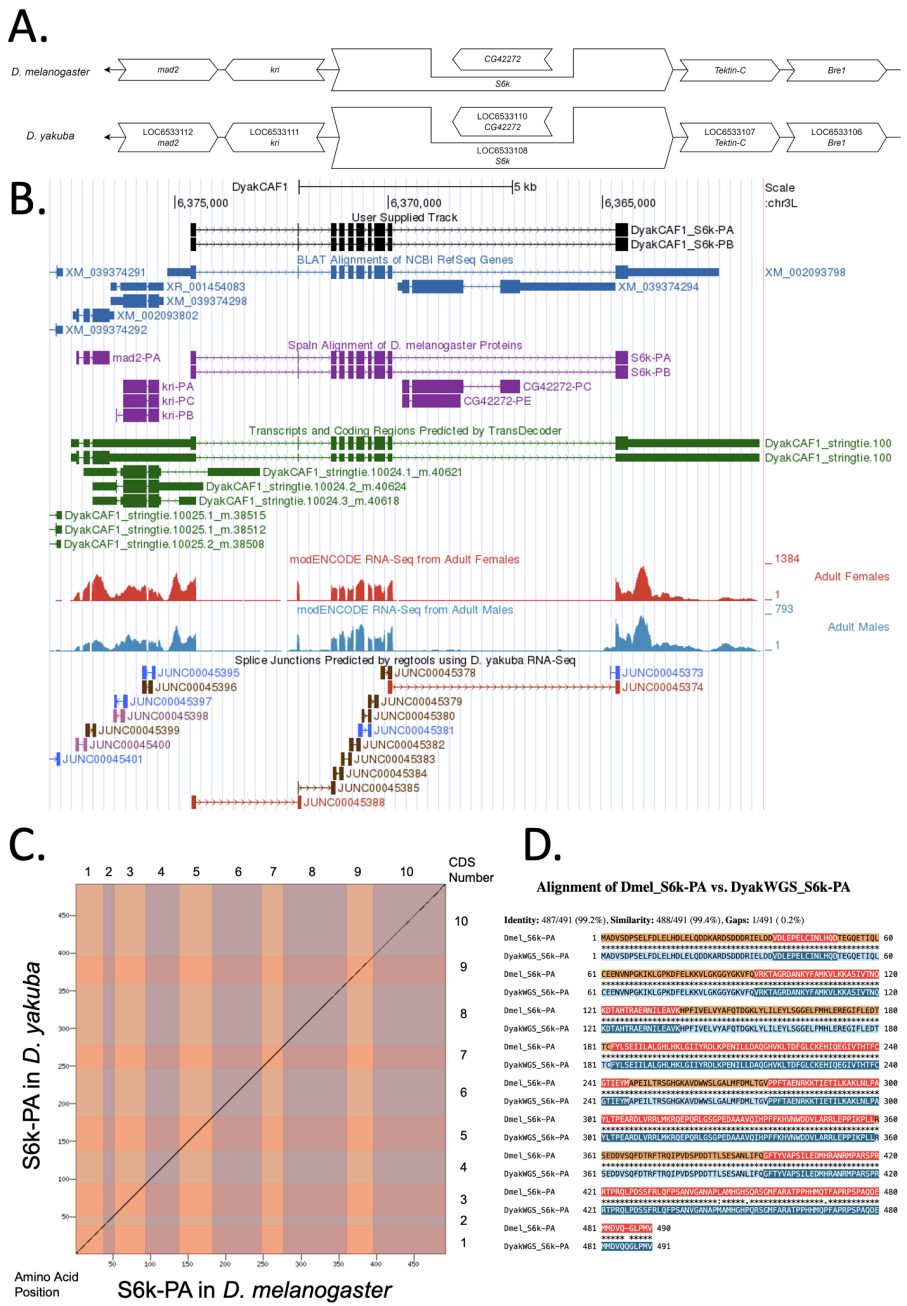
**
(A) Synteny comparison of the genomic neighborhoods for *S6k* in *Drosophila melanogaster* and *D. yakuba* .** Thin underlying arrows indicate the DNA strand within which the target gene– *S6k* –is located in *D. melanogaster* (top) and *D. yakuba* (bottom) genomes. Thin arrows pointing to the left indicate that *S6k* is on the negative (-) strand in *D. yakuba* and *D. melanogaster* . The wide gene arrows pointing in the same direction as *S6k* are on the same strand relative to the thin underlying arrows, while wide gene arrows pointing in the opposite direction of *S6k* are on the opposite strand relative to the thin underlying arrows. White gene arrows in *D. yakuba* indicate orthology to the corresponding gene in *D. melanogaster* . Gene symbols given in the *D. yakuba* gene arrows indicate the orthologous gene in *D. melanogaster* , while the locus identifiers are specific to *D. yakuba* . **(B) Gene Model in GEP UCSC Track Data Hub (Raney et al., 2014).** The coding-regions of *S6k* in *D. yakuba* are displayed in the User Supplied Track (black); CDSs are depicted by thick rectangles and introns by thin lines with arrows indicating the direction of transcription. Subsequent evidence tracks include BLAT Alignments of NCBI RefSeq Genes (dark blue, alignment of Ref-Seq genes for *D. yakuba* ), Spaln of D. melanogaster Proteins (purple, alignment of Ref-Seq proteins from *D. melanogaster* ), Transcripts and Coding Regions Predicted by TransDecoder (dark green), RNA-Seq from Adult Females and Adult Males (red and light blue, respectively; alignment of Illumina RNA-Seq reads from *D. yakuba* ), and Splice Junctions Predicted by regtools using *D. yakuba* RNA-Seq (SRP006203). Splice junctions shown have a read-depth of 10-49, 100-499, 500-999, >1000 supporting reads in blue, pink, brown, and red, respectively. **(C) Dot Plot of S6k-PA in *D. melanogaster* ( *x* -axis) vs. the orthologous peptide in *D. yakuba* ( *y* -axis).** Amino acid number is indicated along the left and bottom; CDS number is indicated along the top and right, and CDS are also highlighted with alternating colors. **(D) Protein alignment between *D. melanogaster* S6k-PA and its putative ortholog in *D. yakuba* .** The alternating colored rectangles represent adjacent CDSs. The symbols in the match line denote the level of similarity between the aligned residues. An asterisk (*) indicates that the aligned residues are identical. A colon (:) indicates the aligned residues have highly similar chemical properties—roughly equivalent to scoring > 0.5 in the Gonnet PAM 250 matrix (Gonnet et al., 1992). A period (.) indicates that the aligned residues have weakly similar chemically properties—roughly equivalent to scoring > 0 and ≤ 0.5 in the Gonnet PAM 250 matrix. A space indicates a gap or mismatch when the aligned residues have a complete lack of similarity—roughly equivalent to scoring ≤ 0 in the Gonnet PAM 250 matrix.

## Description

**Table:** 

*This article reports a predicted gene model generated by undergraduate work using a structured gene model annotation protocol defined by the Genomics Education Partnership (GEP; thegep.org) for Course-based Undergraduate Research Experience (CURE). The following information in this box may be repeated in other articles submitted by participants using the same GEP CURE protocol for annotating Drosophila species orthologs of Drosophila melanogaster genes in the insulin signaling pathway.* "In this GEP CURE protocol students use web-based tools to manually annotate genes in non-model *Drosophila* species based on orthology to genes in the well-annotated model organism fruitfly *Drosophila melanogaster* . The GEP uses web-based tools to allow undergraduates to participate in course-based research by generating manual annotations of genes in non-model species (Rele et al., 2023) . Computational-based gene predictions in any organism are often improved by careful manual annotation and curation, allowing for more accurate analyses of gene and genome evolution (Mudge and Harrow 2016; Tello-Ruiz et al., 2019) . These models of orthologous genes across species, such as the one presented here, then provide a reliable basis for further evolutionary genomic analyses when made available to the scientific community.” (Myers et al., 2024) . “The particular gene ortholog described here was characterized as part of a developing dataset to study the evolution of the Insulin/insulin-like growth factor signaling pathway (IIS) across the genus *Drosophila* . The Insulin/insulin-like growth factor signaling pathway (IIS) is a highly conserved signaling pathway in animals and is central to mediating organismal responses to nutrients (Hietakangas and Cohen 2009; Grewal 2009) .” (Myers et al., 2024) . “ *D. yakuba* (Taxonomic ID: 7245) is part of the *melanogaster* species group within the subgenus *Sophophora* of the genus *Drosophila* (Sturtevant 1939; Bock and Wheeler 1972) . It was first described by Burla (1954). *D. yakuba* is wide-spread in sub-Saharan Africa and Madagascar (Lemeunier et al., 1986; https://www.taxodros.uzh.ch, accessed 1 Feb 2023; Markow and O'Grady 2005) where figs served as a primary host along with other rotting fruits (Lachaise and Tsacas 1983) .” (Koehler et al., 2024) .

We propose a gene model for the *D. yakuba* ortholog of the *D. melanogaster* *Ribosomal protein S6 kinase* ( *S6k* ) gene. The genomic region of the ortholog corresponds to the uncharacterized protein LOC6533108 (RefSeq accession XP_002093834.1 ) in the Dyak_CAF1 Genome Assembly of *D. yakuba* (GenBank Accession: GCA_000005975.1 ). This model is based on RNA-Seq data from *D. yakuba* ( SRP006203 ) and *S6k* in *D. melanogaster* using FlyBase release FB2022_04 ( GCA_000001215.4 ; Larkin et al., 2021).

*Ribosomal protein S6 kinase* ( *S6k* aka p70S6K, FBgn0283472) is part of the insulin signaling pathway downstream of the target of rapamycin ( *dTOR* ) (Toker 2000) , homologous to mammalian p70S6k (Watson et al., 1996) . S6k is a serine/threonine kinase in *Drosophila melanogaster* and acts as a regulator of cell size (Montagne et al., 1999) , as well as innate immunity and senescence (Fabian et al., 2021) . The species summary can be found in the box above.

***Synteny***

The target gene, *S6k* , occurs on chromosome 3L in *D. melanogaster* and the gene *CG42272* ( *CG42272 )* nests within it. *S6k* is flanked upstream by *mad2* ( *mad2 )* and *krishah* ( *kri* ) and downstream by *Tektin C* ( *Tektin-C* ) and *Bre1* ( *Bre1* ). The *tblastn* search of *D. melanogaster* S6k-PA (query) against the *D. yakuba* (GenBank Accession: GCA_000005975.1 ) Genome Assembly (database) placed the putative ortholog of *S6k* within scaffold chromosome 3L (CM000159.2) at locus LOC6533108 ( XP_002093834.1 )— with an E-value of 3e-69 and a percent identity of 43.04%. Furthermore, the putative ortholog of *CG42272* ( LOC6533110 ; XP_039230228.1 ), nests within LOC6533108 (E-value: 0.0; identity: 87.79%, as determined by *blastp* ; Figure 1A, A ltschul et al., 1990) and is flanked upstream by LOC6533112 ( XP_002093838.1 ) and LOC6533111 ( XP_039230232.1 ), which correspond to *mad2* and *kri* in *D. melanogaster* (E-value: 6e-153 and 0.0; identity: 96.62% and 98.08%, respectively, as determined by *blastp* ). The putative ortholog of *S6k* is flanked downstream by LOC6533107 ( XP_002093833.1 ) and LOC6533106 ( XP_002093832.1 ), which correspond to *Tektin-C* and *Bre1* in *D. melanogaster* (E-value: 0.0 and 0.0; identity: 100.00% and 98.28%, respectively, as determined by *blastp* ). The putative ortholog assignment for *S6k* in *D. yakuba* is supported by the following evidence: The genes surrounding the *S6k* ortholog are orthologous to the genes at the same locus in *D. melanogaster* and local synteny is completely conserved, supported by results generated from *blastp* , so we conclude that LOC6533108 is the correct ortholog of *S6k* in *D. yakuba* ( Figure 1A ).

***Protein Model***

*S6k* in *D. yakuba* has two mRNA isoforms ( *S6k-RA; S6k-RB* ) that encode one unique protein-coding isoform (S6k-PA and S6k-PB; Figure 1B ). The gene contains ten CDSs. Relative to the ortholog in *D. melanogaster* , the RNA CDS number and protein isoform count are conserved. The sequence of S6k-PA in *D. yakuba* has 99.19% identity (E-value: 0.0) with the protein-coding isoform S6k-PA in *D. melanogaster* , as determined by *blastp* ( Figure 1D ). Coordinates of this curated gene model of S6k-PA and S6k-PB are stored by NCBI at GenBank/BankIt (accession **BK064475 and BK064476** , respectively **)** . These data are also archived in the CaltechDATA repository (see “Extended Data” section below).

## Methods

Detailed methods including algorithms, database versions, and citations for the complete annotation process can be found in Rele et al. (2023). Briefly, students use the GEP instance of the UCSC Genome Browser v.435 (https://gander.wustl.edu; Kent WJ et al., 2002; Navarro Gonzalez et al., 2021) to examine the genomic neighborhood of their reference IIS gene in the *D. melanogaster* genome assembly (Aug. 2014; BDGP Release 6 + ISO1 MT/dm6). Students then retrieve the protein sequence for the *D. melanogaster* target gene for a given isoform and run it using *tblastn* against their target *Drosophila* species genome assembly (GenBank Accession: GCA_000005975.1 ) on the NCBI BLAST server (https://blast.ncbi.nlm.nih.gov/Blast.cgi; Altschul et al., 1990) to identify potential orthologs. To validate the potential ortholog, students compare the local genomic neighborhood of their potential ortholog with the genomic neighborhood of their reference gene in *D. melanogaster* . This local synteny analysis includes at minimum the two upstream and downstream genes relative to their putative ortholog. They also explore other sets of genomic evidence using multiple alignment tracks in the Genome Browser, including BLAT alignments of RefSeq Genes, Spaln alignment of *D. melanogaster* proteins, multiple gene prediction tracks (e.g., GeMoMa, Geneid, Augustus), and modENCODE RNA-Seq from the target species. Detailed explanation of how these lines of genomic evidenced are leveraged by students in gene model development are described in Rele et al. (2023). Genomic structure information (e.g., CDSs, intron-exon number and boundaries, number of isoforms) for the *D. melanogaster* reference gene is retrieved through the Gene Record Finder (https://gander.wustl.edu/~wilson/dmelgenerecord/index.html; Rele et al *.,* 2023). Approximate splice sites within the target gene are determined using *tblastn* using the CDSs from the *D. melanogaste* r reference gene. Coordinates of CDSs are then refined by examining aligned modENCODE RNA-Seq data, and by applying paradigms of molecular biology such as identifying canonical splice site sequences and ensuring the maintenance of an open reading frame across hypothesized splice sites. Students then confirm the biological validity of their target gene model using the Gene Model Checker (https://gander.wustl.edu/~wilson/dmelgenerecord/index.html; Rele et al., 2023), which compares the structure and translated sequence from their hypothesized target gene model against the *D. melanogaster* reference gene model. At least two independent models for this gene were generated by students under mentorship of their faculty course instructors. These models were then reconciled by a third independent researcher mentored by the project leaders to produce the final model presented here. Note: comparison of 5' and 3' UTR sequence information is not included in this GEP CURE protocol.
